# Supplementary figures and images for: Prenatal exposure to the probiotic Lactococcus lactis decreases anxiety-like behavior and modulates cortical cytoarchitecture in a sex specific manner
Source: PLoS One. 2020 Jul 9;15(7):e0223395. doi: 10.1371/journal.pone.0223395 (PMC7347133; doi:10.1371/journal.pone.0223395)

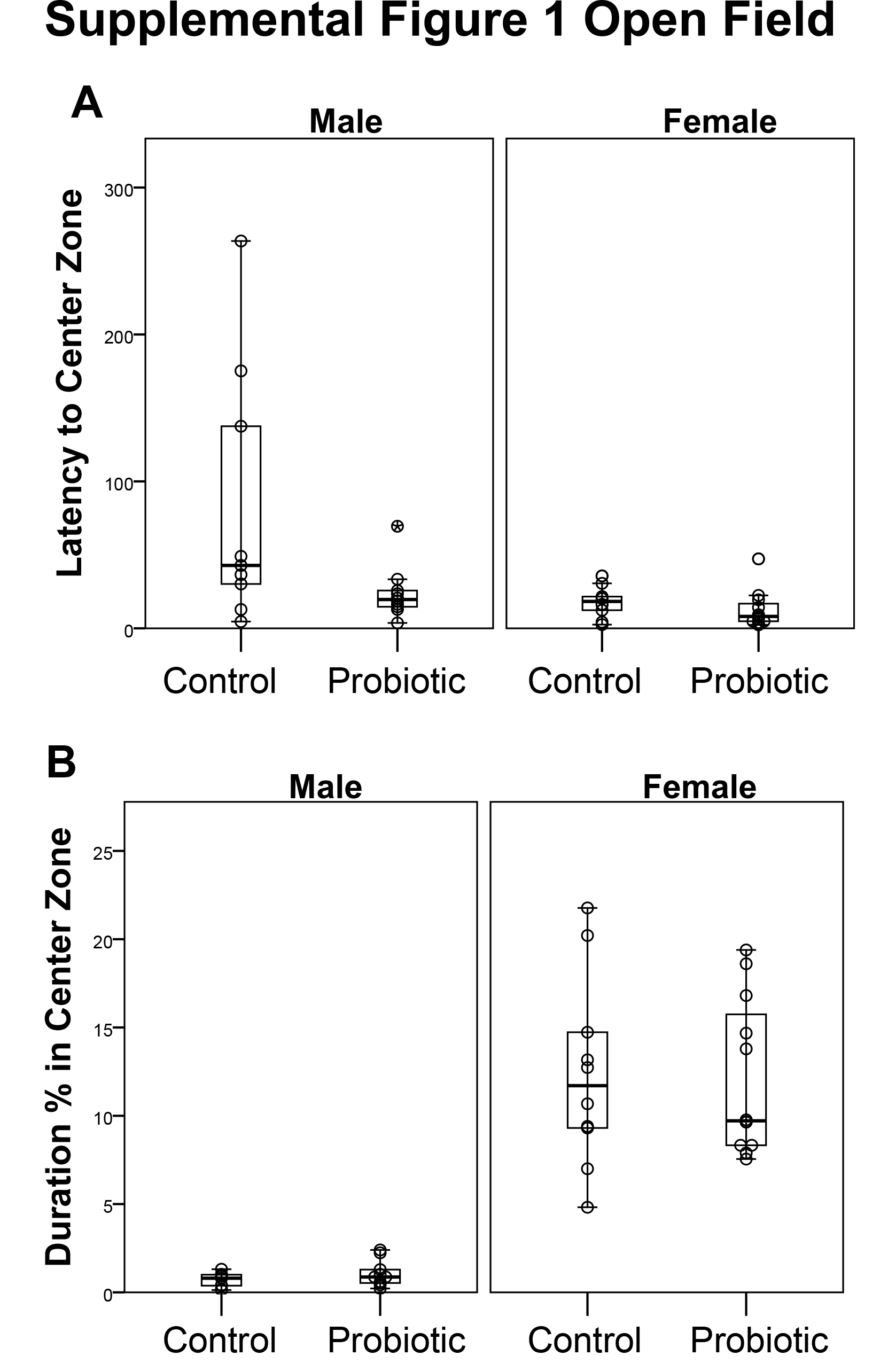

Supplement: S1 Fig — Maternal probiotic supplementation (n = 22) did not have a significant effect on open field behavior, neither on the latency to enter the center zone compared to control mice (n = 18) (A) nor in the time spent in the center (B). We did observe a sex effect, where female mice (n = 22) spend significantly more time in the center than male mice (n = 18) (B). (TIF) [file pone.0223395.s002.tif]

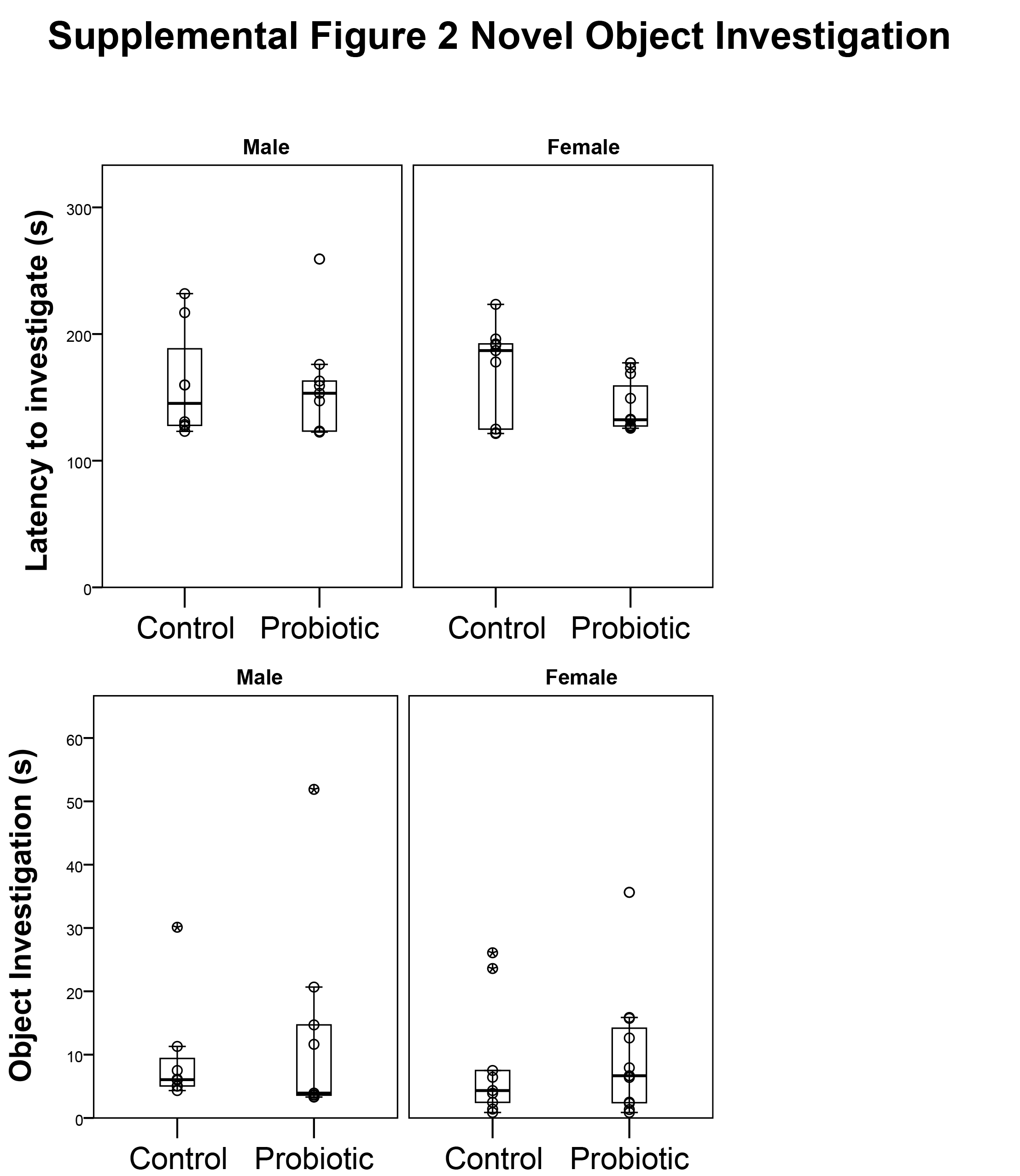

Supplement: S2 Fig — Maternal probiotic supplementation (n = 20) did not have a significant effect on novel object investigation, neither in the latency to approach the object compared to the control mice (n = 17) (A), nor in the time spent investigating the object (B). (TIF) [file pone.0223395.s003.tif]

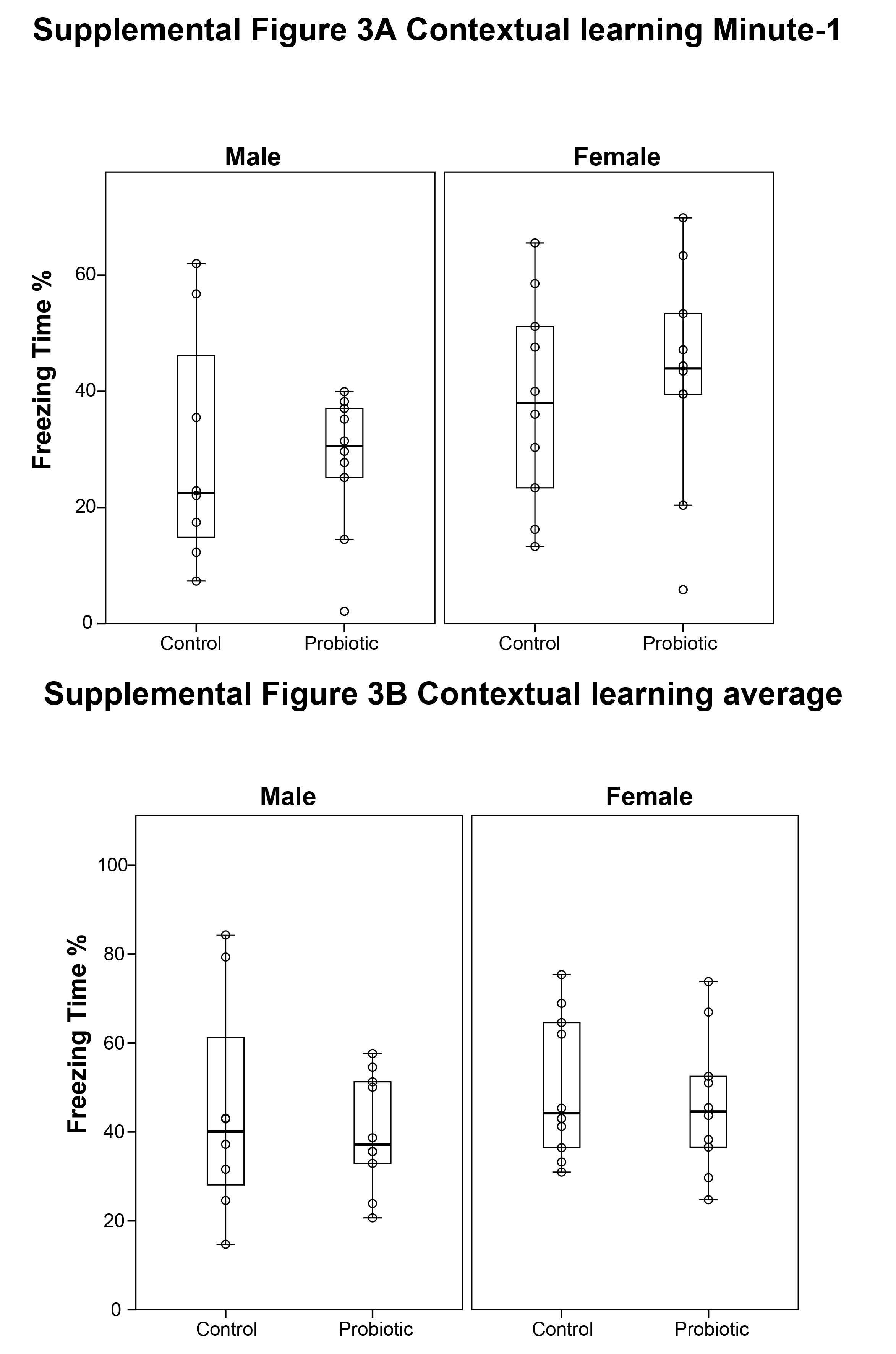

Supplement: S3 Fig — (A) Female mice showed higher levels of freezing in the first minute of contextual learning but there was no treatment effect. (B) Probiotic exposure had no effect on average freezing time during the 5-min session showed no effect of treatment or sex. (TIF) [file pone.0223395.s004.tif]

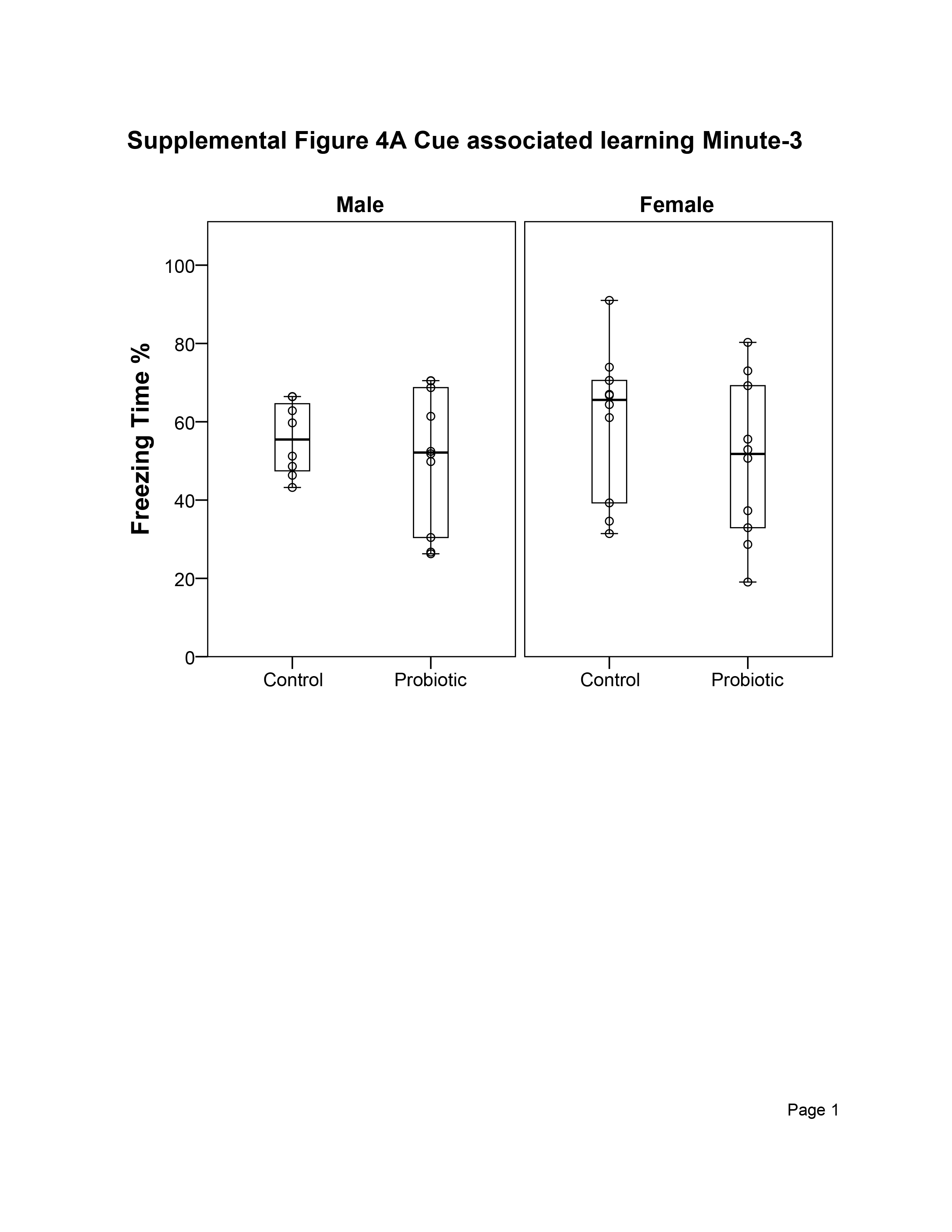

Supplement: S4 Fig — Probiotic exposure did not have an effect on the first minute response to tone in either male (control n = 8 and probiotic n = 10) or female mice (control n = 10 and probiotic n = 10). (TIF) [file pone.0223395.s005.tif]
