# Supplementary material for: Prenatal exposure to the probiotic Lactococcus lactis decreases anxiety-like behavior and modulates cortical cytoarchitecture in a sex specific manner
Source: PLoS One. 2020 Jul 9;15(7):e0223395. doi: 10.1371/journal.pone.0223395 (PMC7347133; doi:10.1371/journal.pone.0223395)
Supplement: Attachment — Submitted filename: Probiotic response letter Plos One 12.12.19.docx [file pone.0223395.s006.docx]

Dear Prof. Homberg,

We are delighted to hear that the Reviewers found our manuscript technically sound, with data supporting the conclusions. We thank the Reviewers for their time and insightful comments. Below, we provide the details of our manuscript revisions, which reflect the Reviewer’s comments and concerns.

**Reviewer comment 1:**

*1-It seems the main aim of the study was not to examine sex dependency of probiotic treatment, so, the Authors need to also focus on the main goal.*

**Author response:**

In agreement with the Reviewer’s comment, we have ensured that the text referring to sex-dependent variation in responses to probiotic treatment does not appear in the Introduction section of the manuscript, but is included in the Discussion (pages 17-18)

**Reviewer comment 2:**

*2-In Abstract, line 34, Authors say "probiotics treatment throughout pregnancy". But according to Methods the treatment occurred during second half of pregnancy.*

**Author response:**

We have corrected this statement in the manuscript Abstract to more accurately reflect the period of probiotic treatment during the second half of pregnancy (line 30).

**Reviewer comment 3:**
*3-While not mothers and not offspring were exposed to stress, a question raise why the Authors introduced the pups to tests that evaluate anxiety stress.*

**Author response:**

Based on the existing literature, linking treatment with probiotic and reduced anxiety (reviewed in Lach et al. Neurotherapeutics 15(1):36-59 2018), we hypothesized that our treatment might result in a decrease of anxiety in the mice. Considering that the behavior in the light-dark box test and in the open field not only is sensible to manipulation increasing anxiety, but also reflect an important variability due to innate higher or lower anxiety levels, we expected these tests to detect an anxiolytic effect of our treatment.

**Reviewer comment 4:**
*4-As far as I know Bifidus (as mentioned in this MS) is not a popular name to replace Bifidobacterium species.*

**Author response:**

We have corrected the text referring to the *Bifidobacterium* species throughout the manuscript.

**Reviewer comment 5:**
*5-Some sentences refer to work of others but no reference is given. For instance, line 56-58 and line 60-61.*

**Author response:**

We have added the appropriate references to the statements included in lines 56-58 and 60-61, and have checked the remainder of the manuscript for proper use of citations.

**Reviewer comment 6:**
*6-I could not understand the dose of probiotic. Further, the Lactobacillus is an anaerobic bacterium. Diluting probiotic in water leads to abrupt decrease of dosage. Therefore, the Authors need to explain how they kept the dosage consistent.*

**Author response:**

In the Materials and Methods section of the manuscript, we mention the dose of the probiotic diluted daily in the drinking water based on the concentration of the powder, which was determined at the beginning and confirmed at the end of the study.

In reference to the probiotic stability in water and the actual dose received by animals - the *Lactobacillus* species are facultative aerobes, so they can tolerate oxygen very well. We therefore did not expect a drastic fast reduction of probiotic concentration in water due to exposure to oxygen.

**Reviewer comment 7:**
*7-In Results Authors must give degree of freedom (F…,…=..) when using ANOVA as they did for light-dark box test. But in some other cases they do not mention F. Moreover, to show a significant difference it is not enough to give just F, but the P values from post test is necessary.*

**Author response:**

Univariate (two-way) ANOVA analysis, with treatment (control and probiotic) and sex (male and female) as factors, was used to analyze some of the data. Both factors have only two groups and no post-hoc analysis is available. For our fear conditioning experiments we ran the repeated measures ANOVA. In the results section we have made sure all the significant results are shown with their p-value and F-value when univariate or repeated measures ANOVA was used for analysis.

**Reviewer comment 8:**
*8- Not necessary to mention (SEM) in Y axis of figures (like fig 2).*

**Author response:**

Some of our figures are shown as means with standard error of means (SEM). For these figures, we would like to keep the SEM in Y-axis as it helps the readers to understand the data distribution. The error bars may also represent standard deviation and we want to make the correct annotation.

**Reviewer comment 9:**
*9- According to “Statistical analyses” error bars indicate SEM. Based on, some comparisons like those in the curves E and F in figures 4 and 5 or curve B in figure 6 seem not to be significant. Please check them.*

**Author response:**

To address the Reviewer’s concern with regards to statistical significance of our findings, we have confirmed the analyses and the P values reported in Figures 4, 5 and 6. In addition, the original data is available at ResearchGate for verification.

**Reviewer comment 10:**
*10-It is not clear how the Authors compared different groups, especially male and female groups. For instance, in many cases it is stated that there was a significant difference between females and males. Question is, in which group, probiotic treated or control group. A comparison of male and female animals in the control group is not valuable, unless the difference is evident between those treated with probiotics. Please clear it.*

**Author response:**

The statistical approach for the analyses was to use a two-way ANOVA with treatment (control and probiotic) and sex (male and female) as factors, this provided us with the statistical mean to identify: main effect of sex (males different from females independently of the treatment), main effect of treatment (control different from probiotic independently of sex) or an interaction of the two (effect of the treatment is dependent of the sex). Thus, when we report a main effect of sex in the manuscript, we report a difference between the males and the females, independently of the treatment effect (e.g. the increased levels of freezing observed in females during the 2^nd^ day of fear conditioning). With this model we also can better understand if any treatment effect is dependent on the sex of the subject (e.g. the difference observed in freezing time during contextual and cue fear training, where the probiotic effect was observed in females only, visible as the decrease of freezing time in treated females compared to control females, while such an effect was not observed in males). We have included the information on which groups were compared in the manuscript text by stating whether the statistical difference referred to a main effect of gender or an interaction of treatment and gender (see examples aforementioned).

**Reviewer comment 11:**

*11-The real Discussion begins from line 383. But from this part on the findings are discussed with just 3 references. Thus, the Authors are expected to present a stronger discussion which also cover all findings.*

**Author response:**

To address this concern, we have revised the Discussion section of the manuscript, which now addresses all the findings and includes appropriate references.

**Reviewer comment 12:**
*12-Despite the MS is almost well written, however, concerning English language, it requires a whole revision.*

**Author response:**

To ensure the manuscript is written in proper English, we have enlisted the help of a native English-speaking scientist in evaluating the manuscript text and suggesting revisions where necessary.

**Reviewer comment 13:**
*13-Please follow consistency over the MS. For instance, Lactobacillus lactis is mentioned differently in lines 73 and 77.*

**Author response:**

We have ensured the consistency of the text referring to *Lactococcus lactis* species used in this study.

**Reviewer comment 14:**
*14-Line 91, what do you mean: (John and Bell 1976, Council 1995).*

**Author response:**

We have ensured that all references cited in the text follow a consistent format.
