# Supplementary material for: Prenatal exposure to the probiotic Lactococcus lactis decreases anxiety-like behavior and modulates cortical cytoarchitecture in a sex specific manner
Source: PLoS One. 2020 Jul 9;15(7):e0223395. doi: 10.1371/journal.pone.0223395 (PMC7347133; doi:10.1371/journal.pone.0223395)
Supplement: Attachment — Submitted filename: Response to Reviewers.docx [file pone.0223395.s007.docx]

Dear Prof. Homberg,

We thank Reviewer 1 for their additional comments. Below, we provide the details of our manuscript revisions, which reflect this Reviewer’s concerns. Revised text within the manuscript is marked in blue font.

**Reviewer’s comment 1:**

*The 3rd question was:
"While not mothers and not offspring were exposed to stress, a question raise why the Authors introduced the pups to tests that evaluate anxiety stress." The Authors say "we hypothesized that our treatment might result in a decrease of anxiety in the mice." However, they research shows that there is no difference between control and probiotic treated animals when testing anxiety.*

**Author’s response:**

We thank the Reviewer for pointing to the potential confusion in the manuscript. Our initial response to the Reviewer was: “Based on the existing literature, linking treatment with probiotic and reduced anxiety (reviewed in Lach et al. Neurotherapeutics 15(1):36-59 2018), we hypothesized that our treatment might result in a decrease of anxiety in mice. Considering that the behavior in the light-dark box test and in the open field not only is sensible to manipulation increasing anxiety, but also reflect an important variability due to innate higher or lower anxiety levels, we expected these tests to detect an anxiolytic effect of our treatment.”

Furthermore, in the Introduction section of our manuscript (pp.3-4) we have emphasized that experimental manipulations affecting the microbiota, such as probiotic treatment or absence of the microbiota, have shown to impact anxiety behaviors in multiple models. Thus, we appear to have addressed the reasons for testing the link between the probiotic treatment and anxiety.

### With respect to the *“difference between control and probiotic-treated animals when testing anxiety”*, we have revised the Results section of our manuscript, titled “Maternal probiotic supplementation reduces anxiety-like offspring behavior in light-dark box test” (pp. 9-11), and a corresponding Figure 1, along with its legend, to highlight the significant findings.

### Specifically, we replaced panel B in the revised Figure 1 with a graph demonstrating the significant impact of probiotic treatment on the distance moved in the light zone in female mice. In summary, revised Figure 1 now demonstrates (A) statistically significant effect of probiotic treatment on the distance moved in the light zone when mice of both sexes are considered in the analyses, (B) statistically significant effect of probiotic treatment on the distance moved in the light zone in female mice, which appear to drive the overall effect, and (C) a trend towards significance for the time spent in the light zone when both sexes are considered (p = 0.059), with probiotic-treated animals spending 10% more time in the light zone. Together with the trend towards significance in the effect of the probiotic treatment on the latency to enter the center zone in the open field test (Figure S1; p = 0.06), our data strongly support an anxiolytic effect of the probiotic treatment.

**Reviewer’s comment 2-1:**

*2- the 7th question was:
"In Results Authors must give degree of freedom (F…,…=..) when using ANOVA as they did for light-dark box test. But in some other cases they do not mention F. Moreover, to show a significant difference it is not enough to give just F, but the P values from post test is necessary."*

**Author’s response:**

To address this concern, we have ensured to include information on both - the degrees of freedom and the p values - in the revised manuscript text and figure legends, where appropriate.

**Reviewer’s comment 2-2:**
*The Authors say "Univariate (two-way) ANOVA analysis, with treatment (control and probiotic) and sex (male and female) as factors, was used to analyze some of the data. Both factors have only two groups and no post-hoc analysis is available." However, suppose you compare only two lines of data, therefore, ANOVA is not a right analysis; in this case an analysis like t-test is more appropriate.*

**Author’s response:**

We thank the Reviewer for their careful consideration of the statistical analyses performed in our manuscript. We agree with the Reviewer that when comparing only two lines of data, a t-test would be more appropriate. However, considering that in addition to the effect of the treatment we also had to consider sex, we were conservative with our data analyses and utilized a method allowing to assess all the important factors, as well as the interactions between these factors. Therefore, ANOVA is an appropriate method of data analysis when multiple factors are taken into consideration.

Importantly, while Reviewer’s comment *“suppose you compare only two lines of data, therefore, ANOVA is not a right analyses; in this case an analysis like a t-test is more appropriate”* is technically correct, there is no difference in the results generated by a t-test and an ANOVA when comparing only two groups - presenting the data from an ANOVA analysis is equivalent to presenting the data from a t-test. Therefore, we feel that to keep the manuscript consistent with regards to data analysis methods, presenting the ANOVA analyses throughout the manuscript is appropriate.

**Reviewer’s comment 3:**
*3-Also: When the between group differences are not statistically significant it means there is no any difference. So, a title like (Maternal probiotic supplementation reduces anxiety-like offspring behavior in light-dark box test) is not acceptable.*

**Author response:**

In our answer to Reviewer’s comment 1 above, we have summarized the significant results of our study in relation to the anxiolytic effects of the probiotic treatment in light-dark box test. Briefly, we have found a significant effect of the probiotic treatment on the distance moved in the light zone (Figure 1A), which is driven by female mice (Figure 1B), and a trend towards significance for the effect of the probiotic treatment on the duration of time spent in the light zone (p = 0.059; Figure 1C). Together, these results justify the title of this manuscript section.
